# Editorial: Women in psychiatry 2022: social psychiatry and psychiatric rehabilitation

**DOI:** 10.3389/fpsyt.2023.1226513

**Published:** 2023-06-06

**Authors:** Helen Killaspy, Armida Mucci

**Affiliations:** ^1^Division of Psychiatry, University College London, London, United Kingdom; ^2^Specialisation School in Psychiatry, University of Campania Luigi Vanvitelli, Caserta, Italy

**Keywords:** women, psychiatry, social, rehabilitation, mental health

Frontiers in Psychiatry's “Women in Psychiatry 2022” initiative aims to promote the work of women scientists across all fields of psychiatry. This Research Topic on Social Psychiatry and Psychiatric Rehabilitation aimed to improve knowledge about the factors influencing mental health outcomes for women, particularly those with more complex mental health problems. We encouraged submissions reporting on studies investigating the relationship between social and psychological factors affecting women's mental health, studies evaluating services or interventions that focus on those with more complex mental health problems, as well as studies investigating the representation of female academics working in this field. Given this fairly broad scope, the papers we included provide an interesting and, perhaps somewhat eclectic, range of subjects.

Two of our included studies investigated whether gender influenced outcomes for people using longer term psychiatric facilities. Rusakovskaya et al. compared social and every day functioning of male and female residents of mental health institutions based in three regions of Russia. Other than gender, participants were similar in sociodemographic characteristics, all had a primary diagnosis of schizophrenia and, on average, they had been living in the facility for over 7 years. The results showed that female residents performed better than their male counterparts on all domains of social and everyday functioning, corroborating previous studies that have identified a poorer prognosis, particularly in regard to functioning, for men diagnosed with schizophrenia. A number of possible explanations for this have been put forward including the earlier age and more insidious onset of the illness amongst males. In Milan, Italy, Cafaro et al. investigated outcomes for people with severe mental illness who participated in an intensive, residential psychiatric rehabilitation program. Males and females had similar diagnostic profiles and scored similarly on ratings of symptoms and functioning at entry to the program. The study found that women were more likely than men to be discharged to independent accommodation, despite having a longer duration of untreated illness. However, they were less likely to have a substance use disorder, which may, in part, explain their better outcomes.

Women have a reputation for being adaptable, as evidenced by Özümerzifon et al. who reported on the feasibility and potential benefits of a virtual dance/movement program conducted during the COVID-19 pandemic for survivors of intimate partner violence. The intervention was originally planned as an in-person program but had to move to an on-line format due to the social distancing restrictions of the pandemic. Nevertheless, 45 women were recruited and randomized and offered either 12 sessions of the program or usual care. The intervention was well received and those who participated in the dance/movement sessions reported improvements in wellbeing and symptoms such as anxiety and post-traumatic stress, as well as benefits from having regular contact with peers.

Moving the focus to women academics, Trimmel et al. investigated the gender of first authors of papers published in three high-impact psychiatry journals over 15 years (from 2004 to 2019). During this period, the percentage of female first authors of papers focusing on the most common three target populations (people with mood disorders, schizophrenia or general mental health problems) increased but male first authors remained in the majority. However, in 2019, over 50% of all original research articles had a female first author and in the two most commonly published psychiatric research fields (biological research and psychosocial epidemiology), the percentage of female first authors was over 50%. These findings suggest that gender equality amongst academics in the mental health field is improving but there is still some way to go. This Research Topic also includes a perspective review by Rexhaj et al. of the literature on caregivers. In keeping with previous research, they identified that around two-thirds of informal care givers are female. Perhaps it is not surprising therefore, that they also found that women academics were more likely than their male counterparts to research in this area.

This overlap between the personal and professional identities of female academics was the focus of the final paper in our Topic—a fascinating study by and Roszik-Volovik et al. who report on how, in the midst of the influx of refugees into Hungary caused by the war in Ukraine, the all female Research Group of Childhood Mental Health, based at Eötvös Loránd University and Semmelweis University in Budapest, transformed itself into a support resource for refugee families. They provided playgroups and activities for the children, many of whom were traumatized by their experiences in Ukraine, that facilitated opportunities for discussions with their parents and specialist advice where needed, and enabled social relationships to form between the refugee families. The experiences of the research group were later evaluated through focus groups, providing insights into the stress of the situation and the process of merging of personal and professional boundaries during this awful and extraordinary moment in history.

We hope you will enjoy reading this collection of papers and, for those of you who are women academics in this or a related field, we hope they provide some inspiration for your own research.

## Author contributions

All authors contributed to this editorial and agreed the final version for publication.

